# Supplementary material for: Structural basis of the interaction between BCL9-Pygo and LDB-SSBP complexes in assembling the Wnt enhanceosome
Source: Nat Commun. 2023 Jun 22;14:3702. doi: 10.1038/s41467-023-39439-9 (PMC10287724; doi:10.1038/s41467-023-39439-9)
Supplement: Supplementary file 1 — Supplementary Information [file 41467_2023_39439_MOESM1_ESM.pdf]

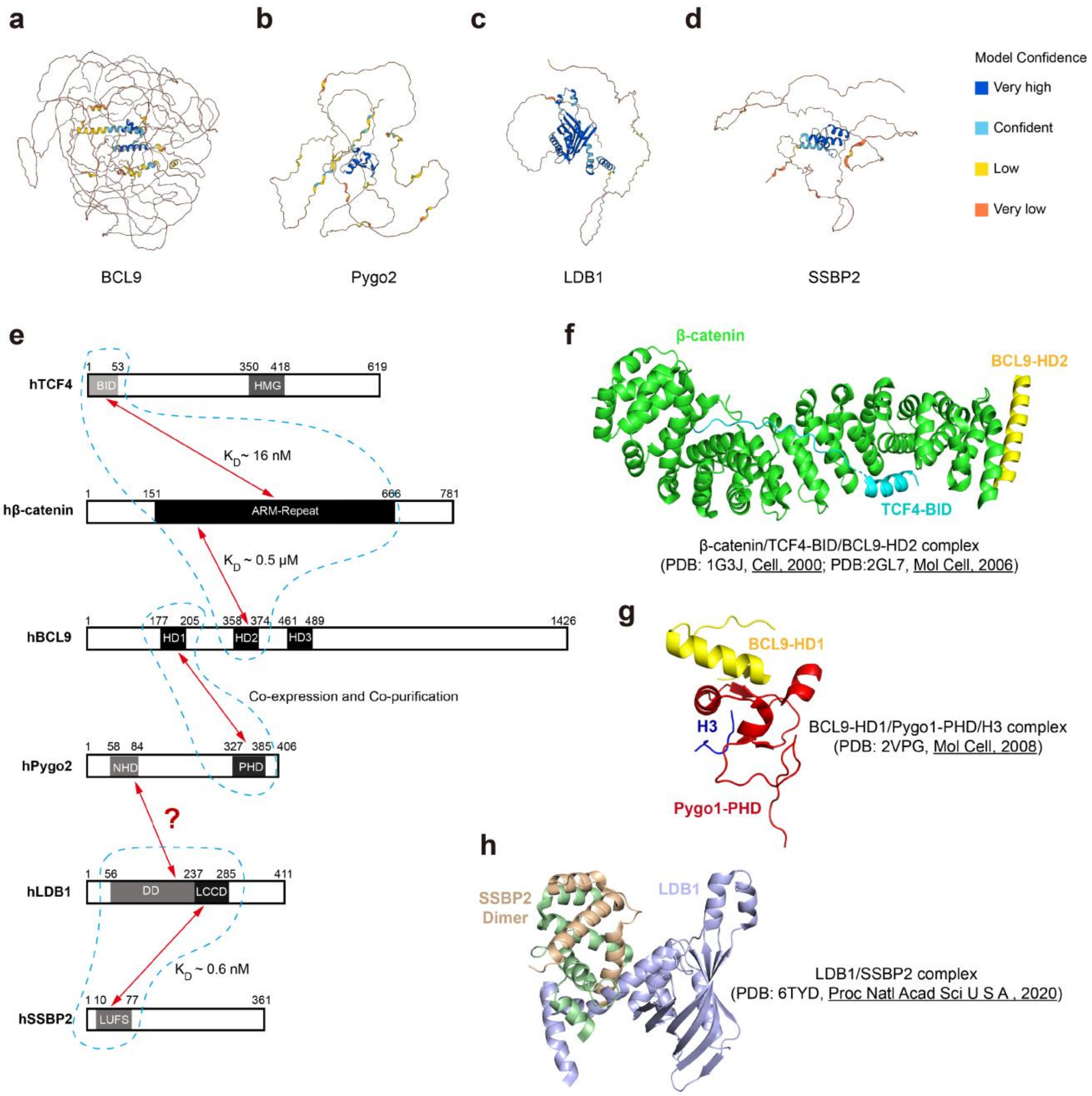

Supplementary Figure 1. Summary of crystal structures and protein-protein interactions between individual components of the human Wnt enhanceosome. Structures predicted by AlphaFold (https://alphafold.com) for full-length BCL9 (**a**), Pygo2 (**b**), LDB1 (**c**) or SSBP2 (**d**). Note that all four proteins are predicted to be largely intrinsically disordered in the absence of binding partners. (**e**) Schematic diagrams of different Wnt enhanceosome components and binding affinities between them (indicated by red arrows) as reported in previous studies[1-4]. Crystal structures of previously determined subcomplexes (marked by blue dashed circles) are shown in (**f**)[2, 5], (**g**)[3] and (**h**)[4], respectively. BID, β-catenin-binding domain; HMG, high mobility group; ARM-repeat, β-catenin Armadillo repeat; HD1/2/3, homology domains 1/2/3; NHD, N-terminal

homology domain; PHD, plant homology domain; DD, dimerization domain; LCCD, LDB/Chip conserved domain; LUFS, LUG/LUH, Flo8 and SSBP conserved domain.

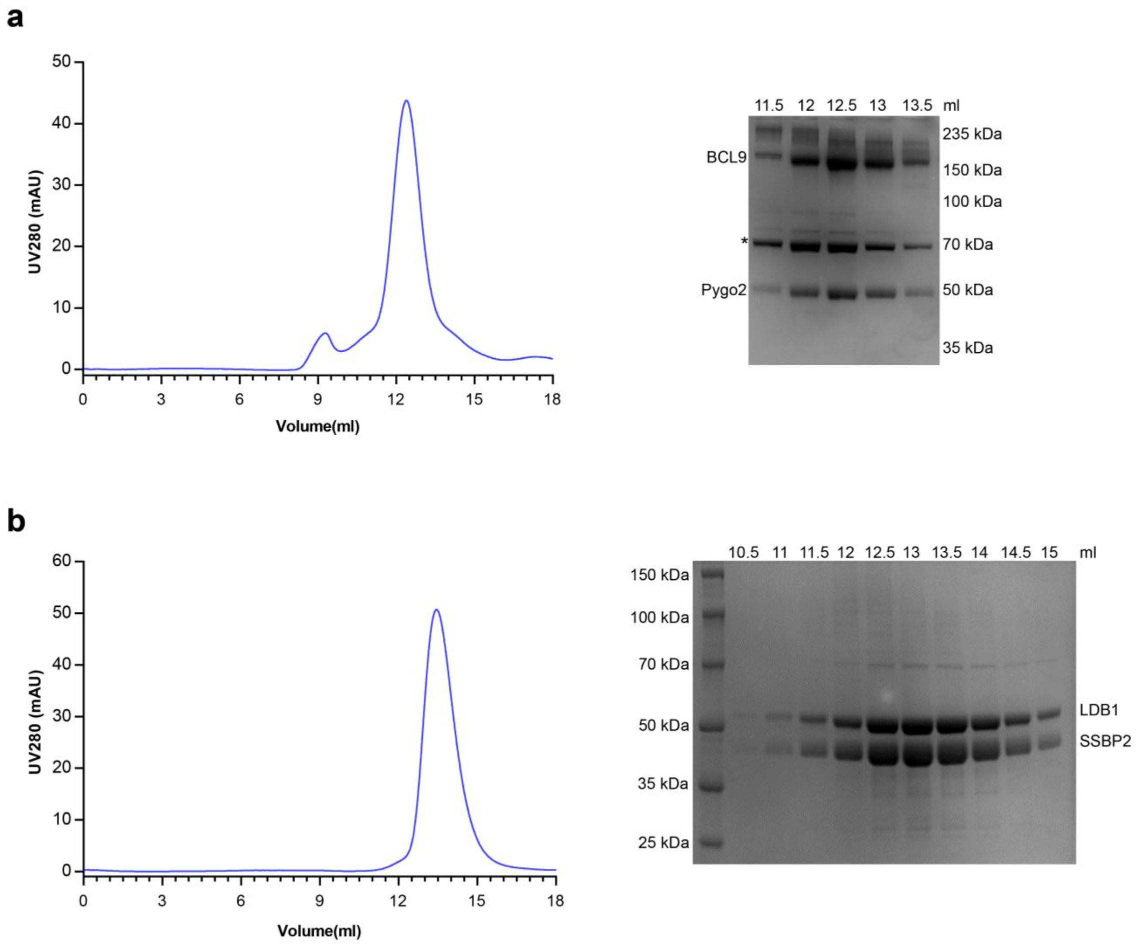

Supplementary Figure 2. Purification of full-length human (**a**) BCL9-Pygo2 or (**b**) LDB1-SSBP2 complex. * indicates contaminating protein chaperone, HSP70. Experiments were independently performed for three times with similar results. Source data are provided as a Source Data file.

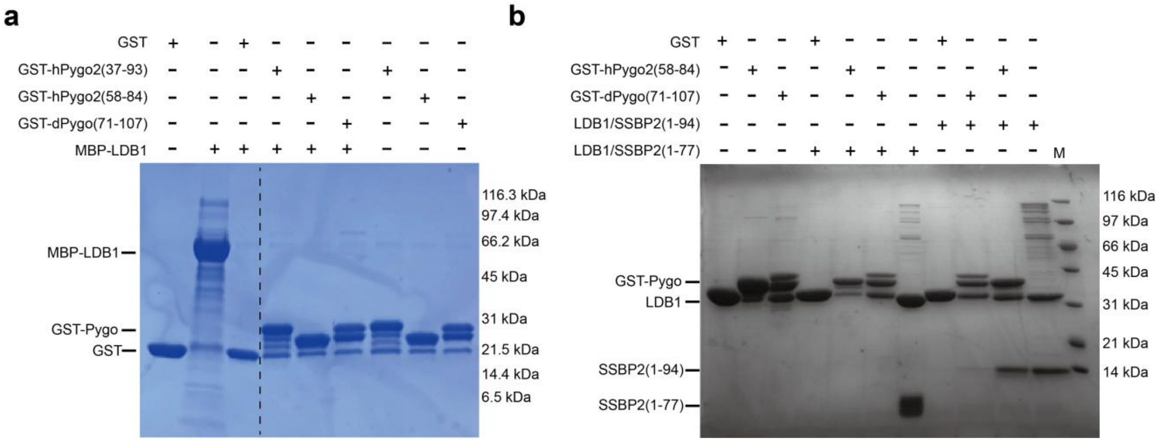

Supplementary Figure 3. Pygo neither binds to (**a**) LDB alone nor (**b**) LDB1(56-285)-SSBP2(1-77), as shown by GST pull-down assays. Experiments were independently performed for three times with similar results. Source data are provided as a Source Data file.

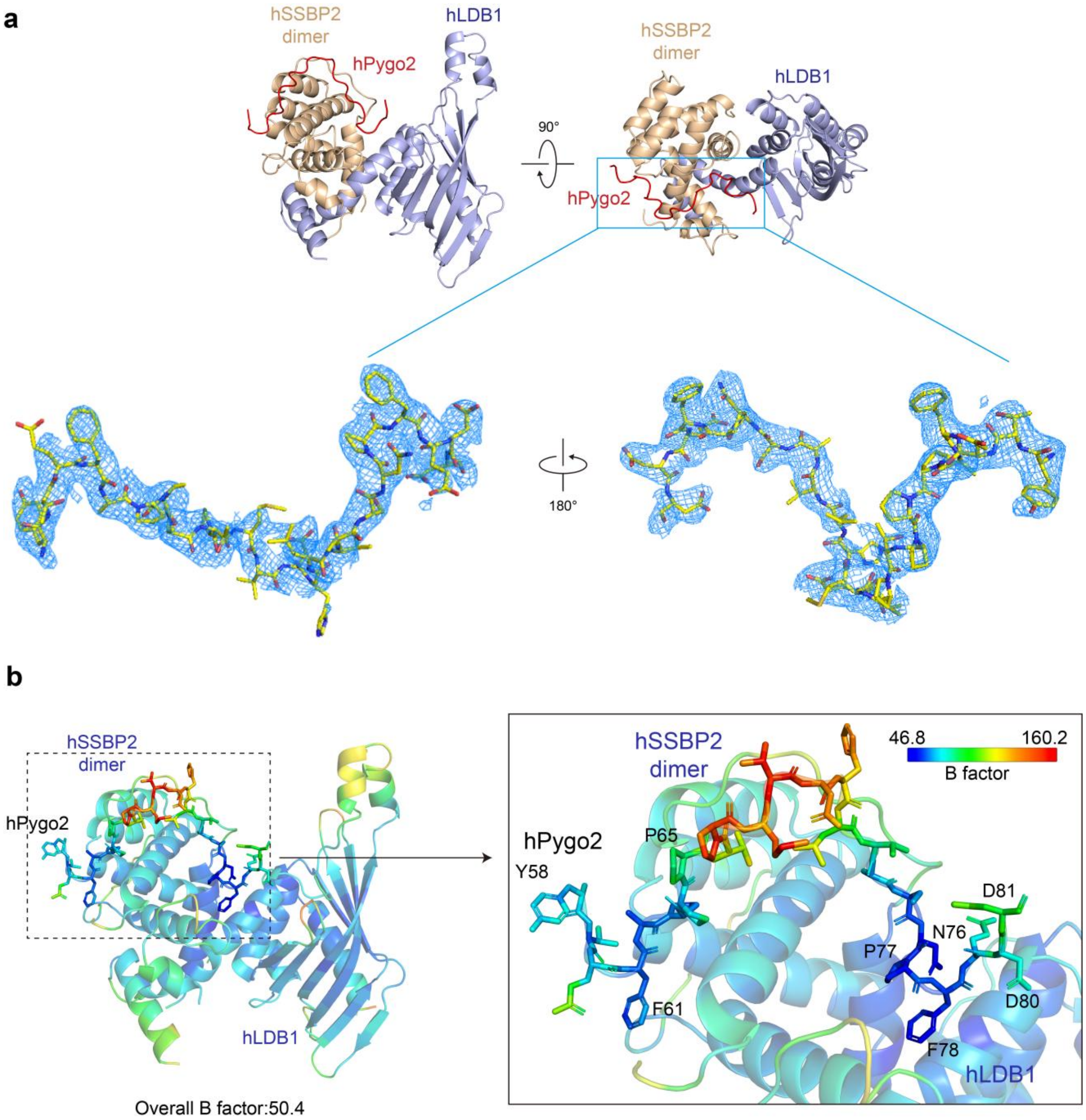

Supplementary Figure 4. (**a**) Pygo2(58-81) with displayed 2Fo-Fc omit map contoured at 1σ level (blue mesh). (**b**) B-factors of Pygo2-NHD atoms in the Pygo2-LDB1-SSBP2 complex crystal structure.

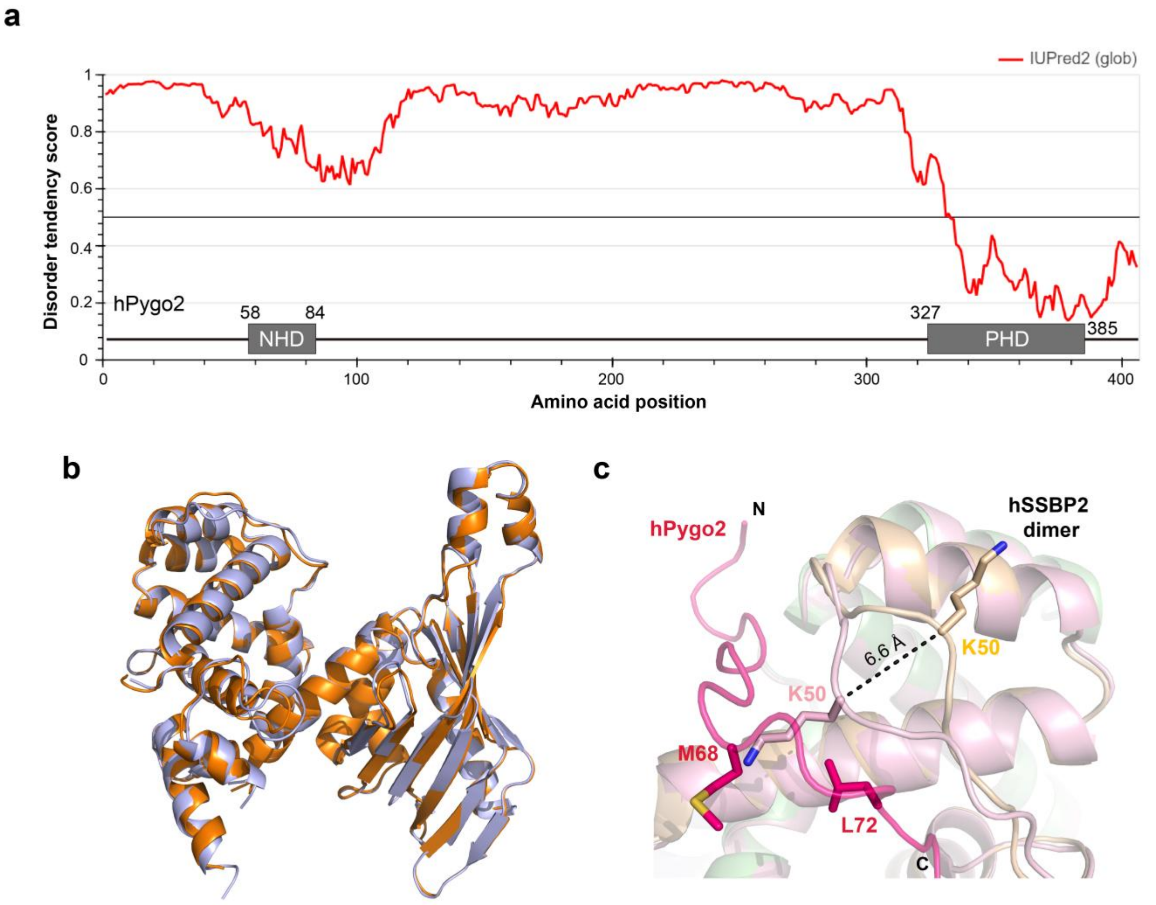

Supplementary Figure 5. Overall structural comparison between LDB1-SSBP2 on its own or as a subcomplex within Pygo2-LDB1-SSBP2. (**a**) Prediction of protein disorder tendency of full-length human Pygo2 by the IUPred2 server (https://iupred2a.elte.hu). The X-axis represents residues 1-406 of full-length human Pygo2, whereas the Y-axis indicates the disorder tendency score for each residue in the context of the Pygo2 sequence whereby sequence stretches with a disorder tendency >0.5 have a high tendency to be structurally disordered. Note that Pygo2-PHD(327-385) is the only sequence stretch predicted to be structured by IUPred2. (**b**) Structural superposition of LDB1-SSBP2 on its own (light blue, 6TYD) or as a subcomplex within Pygo2-LDB1-SSBP2 (orange). (**c**) The conformational difference between the SSBP2 dimer in the Pygo2-LDB1-SSBP2 ternary complex (red, Pygo2; lime, SSBP2 molecule A; wheat, SSBP2 molecule B) and the SSBP2 dimer in the LDB1-SSBP2 subcomplex (pink, 6TYD).

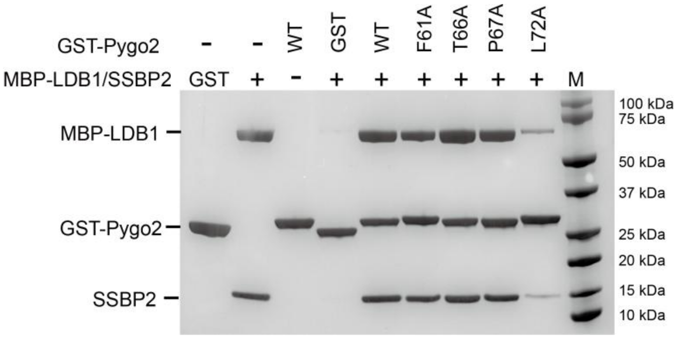

Supplementary Figure 6. MBP pull-down assays between MBP-tagged LDB1, SSBP2 and GST-tagged wt or mutant Pygo2 bearing alanine substitutions of individual interface residues (see also main Fig. 5); GST, negative control. Experiments were independently performed for three times with similar results. Source data are provided as a Source Data file.

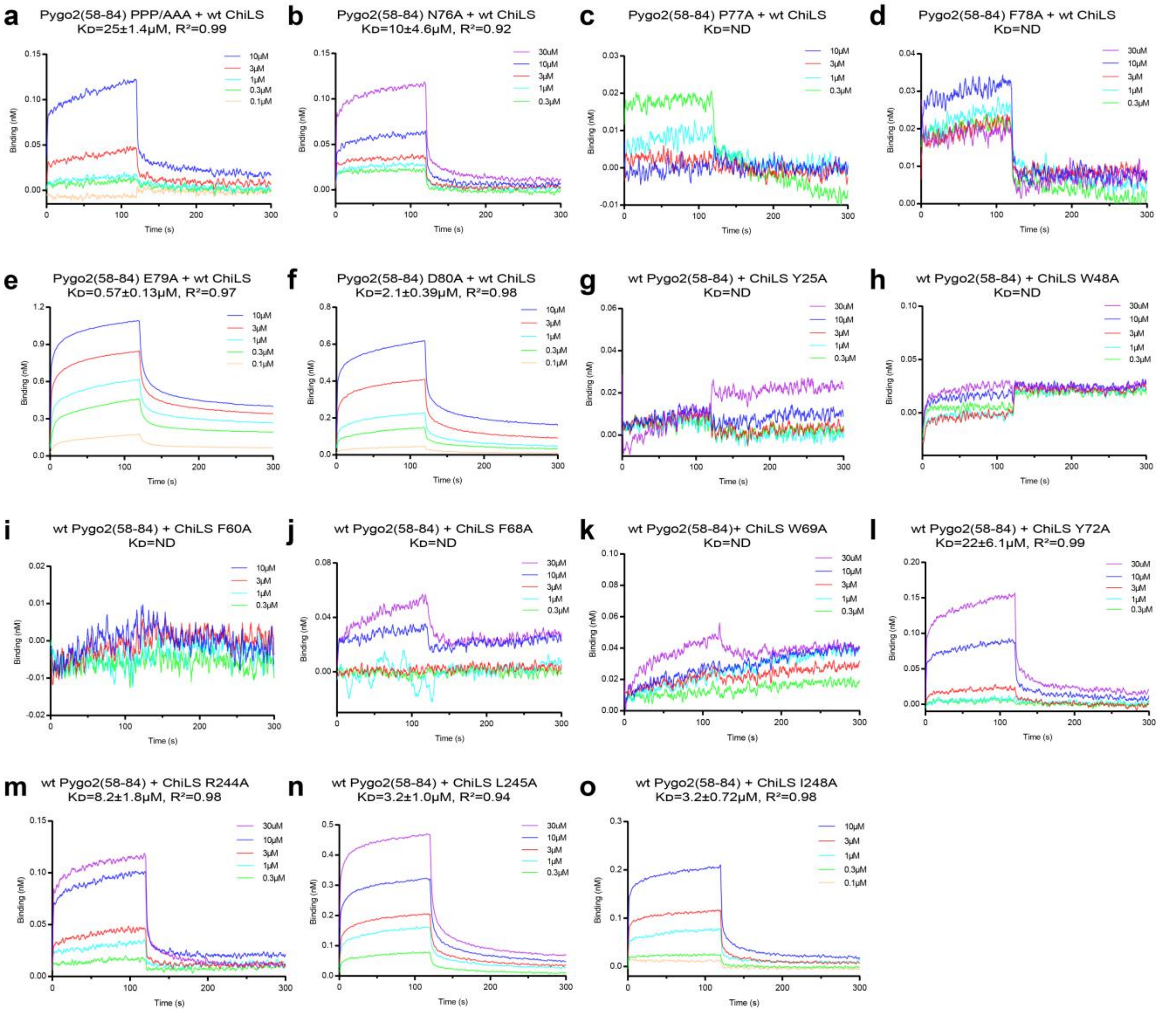

Supplementary Figure 7. Binding affinities between different N-terminal Pygo2 fragments and ChiLS, as measured by BLI assays, (**a-f**) Pygo2(58-84) mutants and wt ChiLS, (**g-l**) wt Pygo2(58-84) and different SSBP2 mutants within ChiLS, (**m-o**) wt Pygo2(58-84) and different LDB1 mutants within ChiLS; ND, no detectable binding. The K_D was calculated based on steady-state analysis and was presented as mean values ± standard deviations (SD). n= 5 of analyzed concentrations, except for Pygo2 P77A and SSBP2 F60A (n= 4 of analyzed concentrations). Source data are provided as a Source Data file.

a

sgRNAs

LDB1 g1	CAGGTATGTAGGCGGATACA
LDB1 g2	GCCATATGGTGTGTGCCTCC
LDB2 g1	TAGGAGGCATACACCATACA
LDB2 g2	CGAAAGGAGAAGAATAGAAG
Pygo1 g1	TCAAATCCATATCTTGGCCC
Pygo1 g2	GGAGCATACTCAGACAATGG
Pygo2 g1	CCCTGCATACTCACATCTGA
Pygo2 g2	GGTGATCCACCATGGGAGTT

Amplification primers

LDB1 F	CATTCAAGCTGTACTCGCCG
LDB1 R	GGAGCATGGTAACGGGTGTT
LDB2 F	TGTGCTCTGCATGGACGTTA
LDB2 R	ATCCTGGCGCTCATTTCCAC
Pygo1 F	TTCACAGGTATTAAGGTAACAGGAC
Pygo1 R	TGAGGTGGATGAGCAGAGGAAT
Pygo2 F	CCTGAGCTGCCCACAGTATC
Pygo2 R	CTGGCTGAGCAAATCGTTGG

Sequencing primers

LDB1 S1	CATTCAAGCTGTACTCGCCG
LDB2 S1	ATCCTGGCGCTCATTTCCAC
Pygo1 S1	TGAGGTGGATGAGCAGAGGAAT
Pygo2 S1	CTGGCTGAGCAAATCGTTGG

b

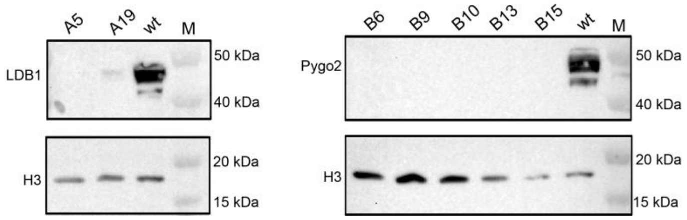

Supplementary Figure 8. Isolation and characterization of LDB1/2 DKO and Pygo1/2 DKO HEK293 cell lines. (**a**) Sequences of gRNAs, amplification and sequencing primers used. Disruptions of LDB1/2 or Pygo1/2 were confirmed by sequencing for each DKO cell line. (**b**) Western blots of lysates from LDB1/2 and Pygo1/2 DKO cell lines, probed with antibody against LDB1 or Pygo2. Anti-histone 3 (H3), loading control. Experiments were independently performed for three times with similar results. Source data are provided as a Source Data file.

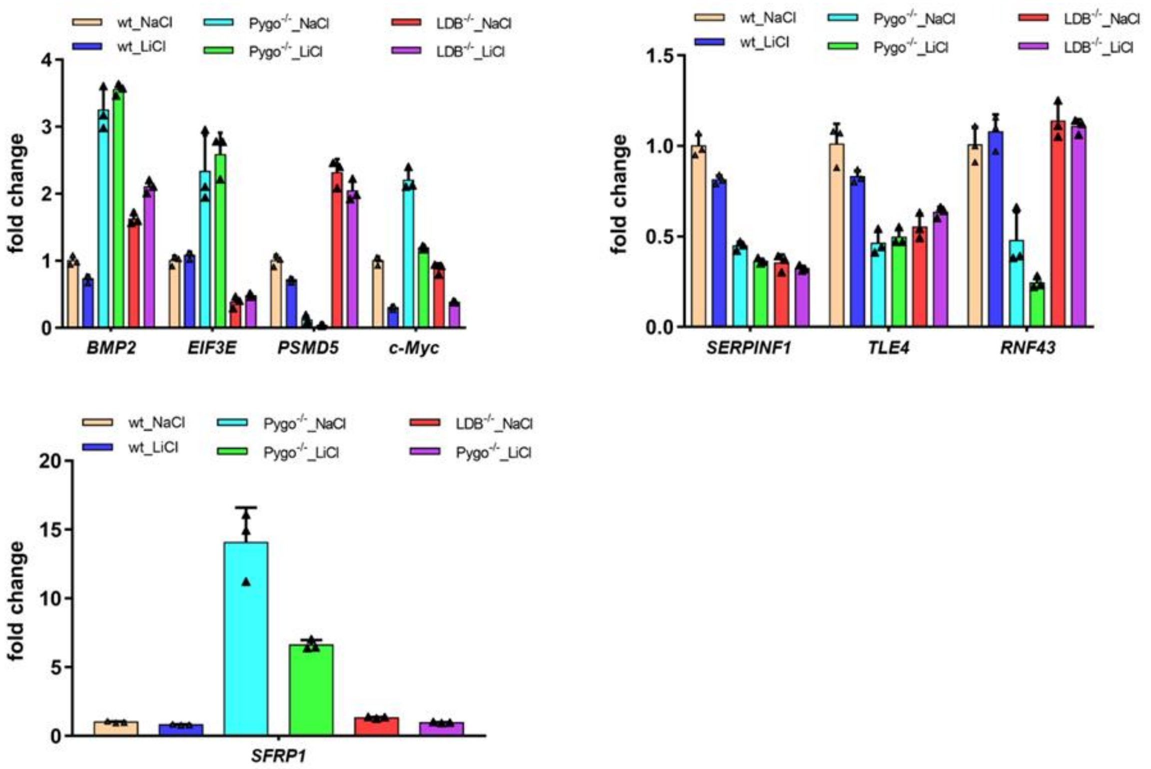

Supplementary Figure 9. RT-qPCR analysis of selected Wnt target genes in wt or DKO HEK293T cells after 6 hours of incubation with ± 20 mM NaCl or LiCl (*ACTB*, reference gene; see also Methods section); expression levels of each gene in control (NaCl) wt HEK293T cells were set to 1. Fold change (Y axis) represents mean values from 3 biologically independent repeats; error bars indicate standard deviations. Source data are provided as a Source Data file.

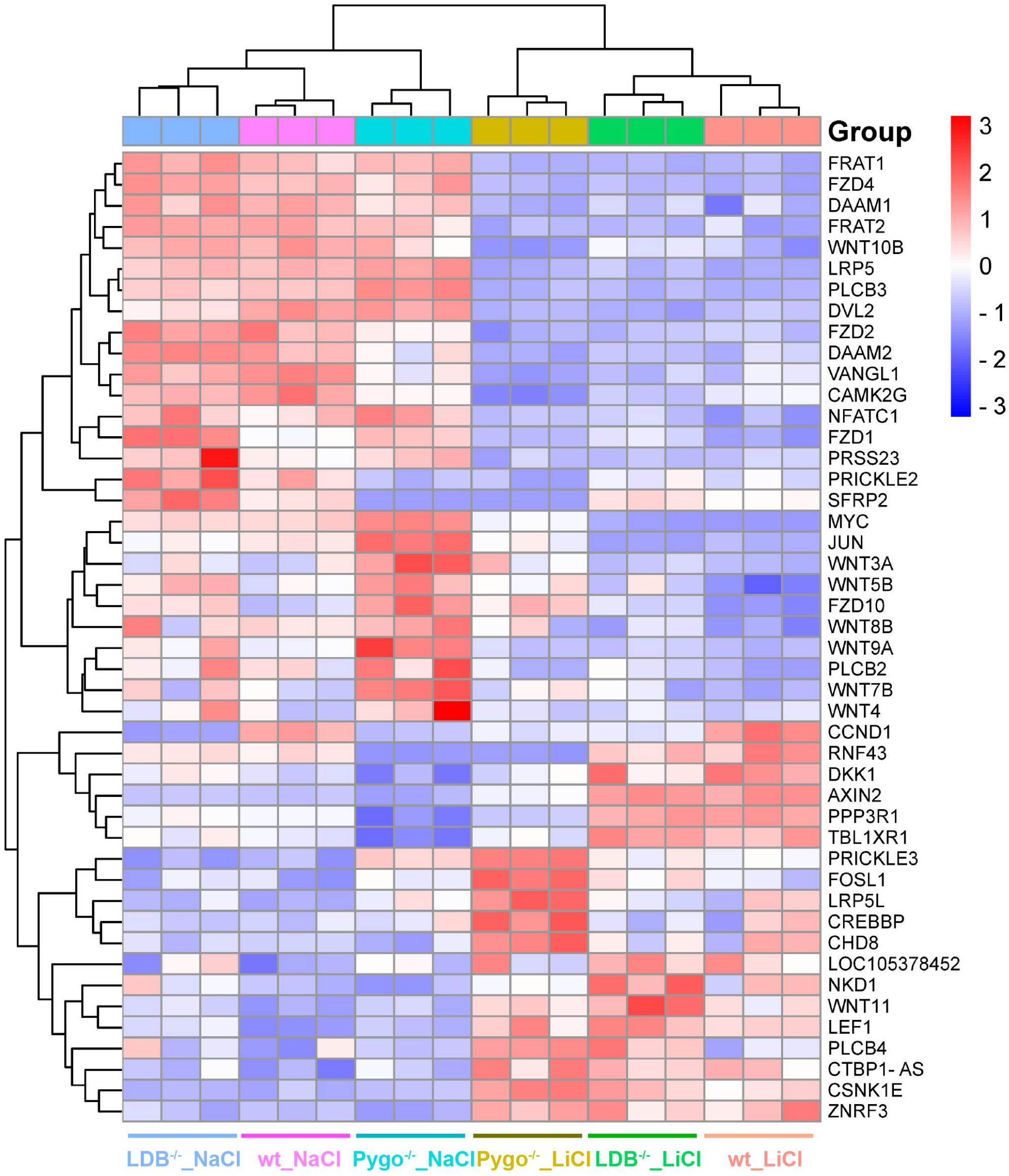

Supplementary Figure 10. Heat-maps based on relative expression levels of differentially expressed genes (DEGs) regulated by Pygo or LDB in response to LiCl stimulation of HEK293T cells (NaCl, control; see also main Fig. 6 and Supplementary Figure 9). DEGs are annotated according to gene ontology (GO) or KEGG (Kyoto Encyclopedia of Genes and Genomes) analysis and include indirect Wnt target genes, some of which may be controlled by Wnt-induced repressors. LDB loss tends to have smaller effects on Wnt target genes than Pygo loss, which may reflect the perdurance of residual LDB protein through numerous cell divisions (e.g. within target chromatin) following the CRISPR-dependent DKO of LDB genes in HEK293T cells. Note that, in

Drosophila, null mutants for *chip* or *pygo* cause equally severe phenotypes, arresting development at the very earliest embryonic stages[25,38], but these phenotypes are only seen if maternal Chip and Pygo are genetically removed as these proteins otherwise perdure through numerous cell divisions, thus providing sufficient function to sustain embryonic development. Source data are provided as a Source Data file.

Supplementary Table 1. Expression levels of selected Wnt target genes

Gene	wt-A	wt-B	PD-A	PD-B	LD-A	LD-B
BMP2	15.52	14.01	28.91	33.20	26.24	32.80
EIF3E	199.51	218.67	243.17	263.56	75.16	87.20
PSMD5	3.77	2.99	0.32	0.20	8.33	6.58
c-Myc	36.28	12.53	48.44	27.76	35.86	13.76
SERPINF1	39.71	35.95	14.79	11.93	19.19	15.36
TLE4	32.18	35.12	9.09	12.32	20.79	23.48
RNF43	2.34	3.24	0.61	0.67	2.34	2.69
SFRP1	5.26	3.87	35.32	23.40	6.93	4.77

wt, wt HEK293T cells; PD, Pygo$^{-/-}$ HEK293T cells; LD: LDB$^{-/-}$ HEK293T cells. “A” or “B” denote cells incubated with 20 mM NaCl (A) or LiCl (B) for 6 hours prior to cell lysis. Numbers represent average FPKM values of 3 independent experiments (see also Methods section). Source data are provided as a Source Data file.

Supplementary Table 2. RT-qPCR Primers for selected Wnt target genes

Gene	Primer sequence (5'-3')	Size (bp)
BMP2	F: GCTTCCACCATGAAGAATCTTTG<br>R: GTGATAAACTCCTCCGTGGG	106
EIF3E	F: GAGGGAATGTGAATCAGTGC<br>R: TGGCATCCAGTCTTGCATTT	212
PSMD5	F: CTCACTCTTTCCCAGATTGGAAG<br>R: CAGTCCAGCTTGGGTTAGTG	174
c-Myc	F: AGCGACTCTGAGGAGGAACA<br>R: GGATAGTCCTTCCGAGTGGA	212
SERPINF1	F: GATCTCAGCTGCAAGATTGC<br>R: CTTGGTGACTTCGCCTTCAT	210
TLE4	F: CAGCTCGACTGTCTGAACAG<br>R: AGCATGAGAAGCAGACCTTG	211
RNF43	F: CATGTTCAACATCACAGAGGGAG<br>R: GATGCTGGCGAATGAGGTG	101
SFRP1	F: GCGAGTTTGCACTGAGGAT<br>R: AGTACTGGCTCTTCACCTTG	225
ACTB	F: TCCTTCCTGGGCATGGAGT<br>R: CGATCCACACGGAGTACTTG	232

Supplementary Table 3. Primer information for construct cloning

Construct	Primer sequence (5'-3')
PCAGGS_BCL9_Flag	F: gcaaagaattcgccaccatgATGCATTCCAGTAACCCT<br>R: tcgtctttgtagtccgagccAAACATCATGTTTCCTGGG
PCAGGS_Pygo2_Flag	F: gcaaagaattcgccaccatgATGGCAGCATCAGCTCCTC<br>R: tcgtctttgtagtccgagccTCCATCGTTTGCTGCCACAA
PCAGGS_Pygo2_Flag NPF/AAA	F: GTTGCATCCGCAGCTGCAGAAGATGACTTCGGAG<br>R: CTCCGAAGTCATCTTCTGCAGCTGCGGATGCAAC
PCAGGS_LDB1_Flag	F: gcaaagaattcgccaccatgATGAGCGTTGGTTGCGCG<br>R: tcgtctttgtagtccgagccCTGGCTCGCTTGGCTGGT
PCAGGS_LDB1_Flag R244A	F: ACCCTGAACTACCTGGCACTGTGCGTGATTCTG<br>R: CAGAATCACGCACAGTGCCAGGTAGTTCAGGGT
PCAGGS_SSBP2_Flag	F: gcaaagaattcgccaccatgATGTACGGCAAAGGCAAG<br>R: tcgtctttgtagtccgagccCACGCTCATTGTCATGCTAG
pGEX-4T-1_dPygo(71-107)	F: aaaacctgtattttcagggcCAGCAGGCGCCACCCACA<br>R: cgacccgggaattccggttattaCGCCGACACCTGGGGACT
pGEX-4T-1_hPygo2(37-93)	F: aaaacctgtattttcagggcCAAATGAAGAGTCCAGAAAAG<br>R: cgacccgggaattccggttattaTGGAGGGGCTGCAACCC
pGEX-4T-1_hPygo2(35-84)	F: aaaacctgtattttcagggcGGCCTGCAAATGAAATCCCC<br>R: cgacccgggaattccggttattaCGCACCGAAATCGTCCTCA
pGEX-4T-1_hPygo2(58-84)	F: aaaacctgtattttcagggcCTGACGGAGTTTGCACCACC<br>R: cgacccgggaattccggttattaGGCTCCGAAGTCATCTTCA
pGEX-4T-1_hPygo2(66-81)	F: aaaacctgtattttcagggcTATCCCATGGTGGATCACCTGG<br>R: cgacccgggaattccggttattaGTCATCTTCAAAAGGGTTG

(Supplementary Table 3, continued)	
pETDuet-1_MBP-LDB1(56-285) and His-SSBP2(1-94) (in short, LDB1-SSBP2)	F1: gccatcaccatcatcaccacATGAAAATCGAAGAAGGTAAACTGGTAATCTGG<br>R1: ctcgaattcggatcctggctTTATTCCGCCGGCGGAGC<br>F2: tataagaaggagatatacatatgCATCACCATCATCACCACGAAAACCTGTATTTTCAGG<br>R2: gatatccaattgagatctgcCTAGTAATCATGGAAGGC
pETDuet-1_MBP-LDB1(56-285) and His-SSBP2(1-77)	F1: gccatcaccatcatcaccacATGAAAATCGAAGAAGGTAAACTGGTAATCTGG<br>R1: ctcgaattcggatcctggctTTATTCCGCCGGCGGAGC<br>F2: tataagaaggagatatacatatgCATCACCATCATCACCACGAAAACCTGTATTTTCAGG<br>R2: gatatccaattgagatctgcTTACTCTGGAGCTGCACAG
His-MBP-LDB1(56-287)	F: tgttccaggggcccggatccGGCATCGGTCGTCACACC<br>R: tgtcgacggagctcgaattcTTATTCCGCCGGCGGAGC
pGEX-4T-1_hPygo2(58-84) F61A	F: CAGGGCTATACGGAGGCAGCACCACCCCCAAC<br>R: GTTGGGGGTGGTGCTGCCTCCGTATAGCCCTG
pGEX-4T-1_hPygo2(58-84) P63A	F: GCTATACGGAGTTTGCAGCACCCCCAACTCCC<br>R: GGGAGTTGGGGGTGCTGCAAACTCCGTATAGC
pGEX-4T-1_hPygo2(58-84) PP63AA	F: GCTATACGGAGTTTGCAGCAGCCCCAACTCCCATGG<br>R: CCATGGGAGTTGGGGCTGCTGCAAACTCCGTATAGC
pGEX-4T-1_hPygo2(58-84) PPP63AAA	F: CTATACGGAGTTTGCAGCAGCCGCAACTCCCATGGTGGAT<br>R: ATCCACCATGGGAGTTGCGGCTGCTGCAAACTCCGTATAG
pGEX-4T-1_hPygo2(58-84) T66A	F: TTGCACCACCCCCAGCACCCATGGTGGATCAC<br>R: GTGATCCACCATGGGTGCTGGGGGTGGTGCAA
pGEX-4T-1_hPygo2(58-84) P67A	F: CACCACCCCCAACTGCAATGGTGGATCACCTG<br>R: CAGGTGATCCACCATTGCAGTTGGGGGTGGTG

(Supplementary Table 3, continued)

pGEX-4T-1_hPygo2(58-84) L72A	F: CCCATGGTGGATCACGCAGTTGCATCCAACCCT<br>R: AGGGTTGGATGCAACTGCGTGATCCACCATGGG
pGEX-4T-1_hPygo2(58-84) N76A	F: CACCTGGTTGCATCCGCACCTTTTGAAGATGACTT<br>R: AAGTCATCTTCAAAAGGTGCGGATGCAACCAGGTG
pGEX-4T-1_hPygo2(58-84) P77A	F: CTGGTTGCATCCAACGCTTTTGAAGATGACTTC<br>R: GAAGTCATCTTCAAAAGCGTTGGATGCAACCAG
pGEX-4T-1_hPygo2(58-84) F78A	F: GTTGCATCCAACCCTGCAGAAGATGACTTCGGAG<br>R: CTCCGAAGTCATCTTCTGCAGGGTTGGATGCAAC
pGEX-4T-1_hPygo2(58-84) E79A	F: GCATCCAACCCTTTTGCAGATGACTTCGGAGC<br>R: GCTCCGAAGTCATCTGCAAAAGGGTTGGATGC
pGEX-4T-1_hPygo2(58-84) D80A	F: TCCAACCCTTTTGAAGCTGACTTCGGAGCCTA<br>R: TAGGCTCCGAAGTCAGCTTCAAAAGGGTTGGA
pGEX-4T-1_hPygo2(58-84) D81A	F: CAACCCTTTTGAAGATGCATTCGGAGCCTAA<br>R: TTAGGCTCCGAATGCATCTTCAAAAGGGTTG
LDB1-SSBP2 Y25A	F: GGAGAAGTTAGCACTCGCAGTATATGAATATCTGCTC<br>R: GAGCAGATATTCATATACTGCGAGTGCTAACTTCTCC
LDB1-SSBP2 W48A	F: ATTTTTATCAGAGATAAGAGCAGAAAAAAACATCACATTG<br>R: CAATGTGATGTTTTTTTCTGCTCTTATCTCTGATAAAAAT
LDB1-SSBP2 F60A	F: GGGAACCACCAGGAGCATTACATTCTTGGTGGTG<br>R: CACCACCAAGAATGTAATGCTCCTGGTGGTTCCC
LDB1-SSBP2 W64A	F: CAGGATTCTTACATTCTGCATGGTGTGTATTTTGGG<br>R: CCCAAAATACACACCATGCAGAATGTAAGAATCCTG

(Supplementary Table 3, continued)

LDB1-SSBP2 F68A	F: CATTCTTGGTGGTGTGTAGCATGGGATCTCTACTGTGCA<br>R: TGCACAGTAGAGATCCCATGCTACACACCACCAAGAATG
LDB1-SSBP2 W69A	F: CTTGGTGGTGTGTATTTGCAGATCTCTACTGTGCAG<br>R: CTGCACAGTAGAGATCTGCAAATACACACCACCAAG
LDB1-SSBP2 Y72A	F: TGTGTATTTTGGGATCTCGCATGTGCAGCTCCAGAGAGA<br>R: TCTCTCTGGAGCTGCACATGCGAGATCCCAAAATACACA
LDB1 N237A -SSBP2	F: GTTGCGGTCTGAGCGCAAGCACCCTGAACTAC<br>R: GTAGTTCAGGGTGCTTGCGCTCAGACCGCAAC
LDB1 N241A -SSBP2	F: GCAACAGCACCCTGGCATACCTGCGTCTGTGC<br>R: GCACAGACGCAGGTATGCCAGGGTGCTGTTGC
LDB1 R244A -SSBP2	F: ACCCTGAACTACCTGGCACTGTGCGTGATTCTG<br>R: CAGAATCACGCACAGTGCCAGGTAGTTCAGGGT
LDB1 L245A -SSBP2	F: CTGAACTACCTGCGTGCATGCGTGATTCTGGAG<br>R: CTCCAGAATCACGCATGCACGCAGGTAGTTCAG
LDB1 I248A -SSBP2	F: TGCGTCTGTGCGTGGCACTGGAGCCGATGCA<br>R: TGCATCGGCTCCAGTGCCACGCACAGACGCA
PCAGGS_BCL9_Avi_Flag	F: gcaaagaattcgccaccatgATGCATTCCAGTAACCCT<br>R: tcgtctttgtagtcacttccTTCGTGCCATTCGATTTTCTGAGCC
PCAGGS_BCL9$^{\triangle HD1}$_Avi_Flag	F: AGCCAAAGTGCAGAACATTTCTAACAACAAGAC<br>R: AAATGTTCTGCACTTTGGCTGGAGTCTTC
PCAGGS_BCL9$^{\triangle HD3}$_Avi_Flag	F: AGGACCCGACGTGGTTGTCCAGCAGTGTTC<br>R: GGACAACCACGTCGGGTCCTATGGTCCC

Note: Sequences from plasmid vectors are shown in lowercase, and sequences from target genes are shown in uppercase.

Supplementary References

1. Sun, J. & Weis, W.I. Biochemical and structural characterization of β-catenin interactions with nonphosphorylated and CK2-phosphorylated Lef-1. *Journal of molecular biology* **405**, 519-530 (2011).
2. Sampietro, J. et al. Crystal structure of a beta-catenin/BCL9/Tcf4 complex. *Molecular cell* **24**, 293-300 (2006).
3. Fiedler, M. et al. Decoding of methylated histone H3 tail by the Pygo-BCL9 Wnt signaling complex. *Molecular cell* **30**, 507-518 (2008).
4. Wang, H. et al. Crystal structure of human LDB1 in complex with SSBP2. *Proceedings of the National Academy of Sciences of the United States of America* **117**, 1042-1048 (2020).
5. Graham, T.A., Weaver, C., Mao, F., Kimelman, D. & Xu, W. Crystal structure of a beta-catenin/Tcf complex. *Cell* **103**, 885-896 (2000).

Uncropped scans of all gels and blots presented in Supplementary Figures

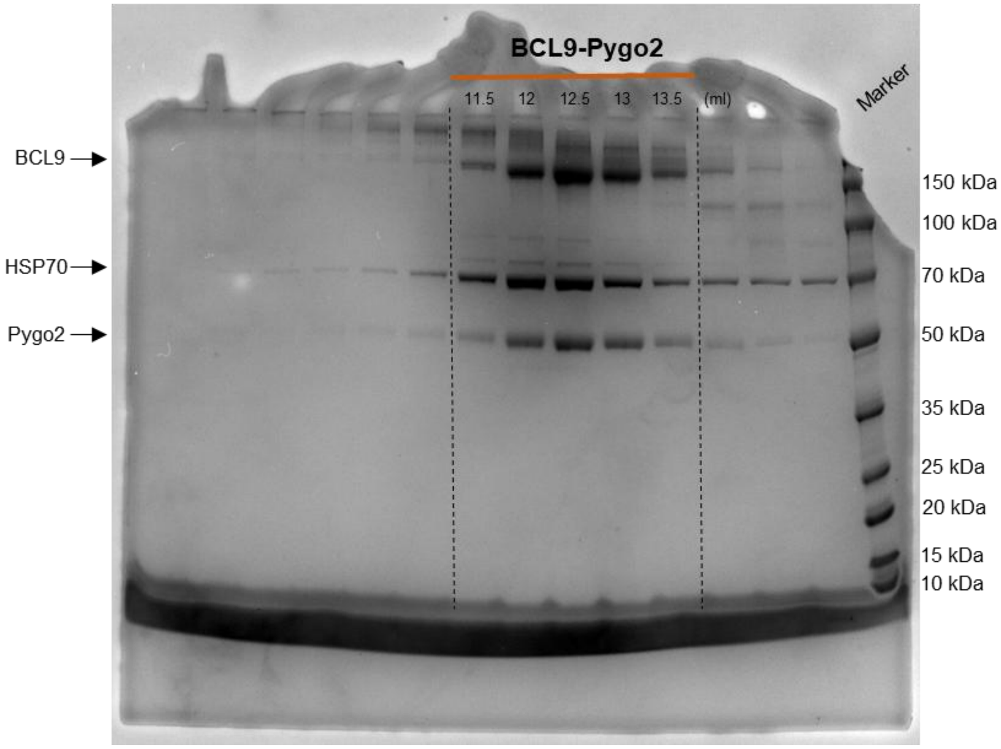

Supplementary Figure 2a

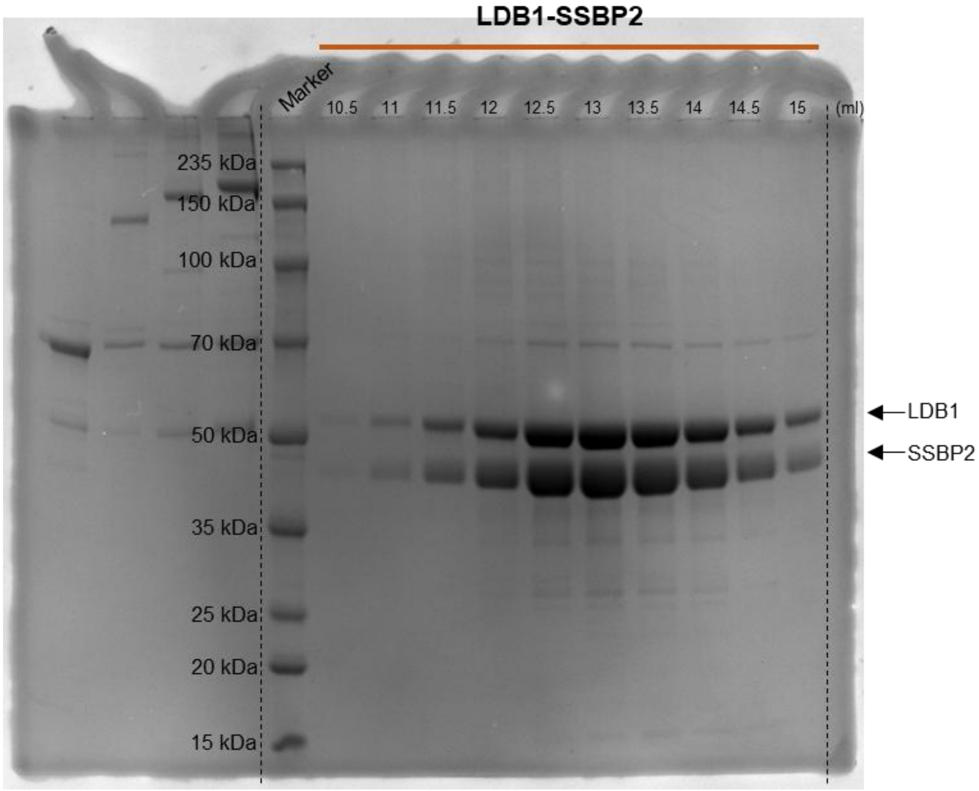

Supplementary Figure 2b

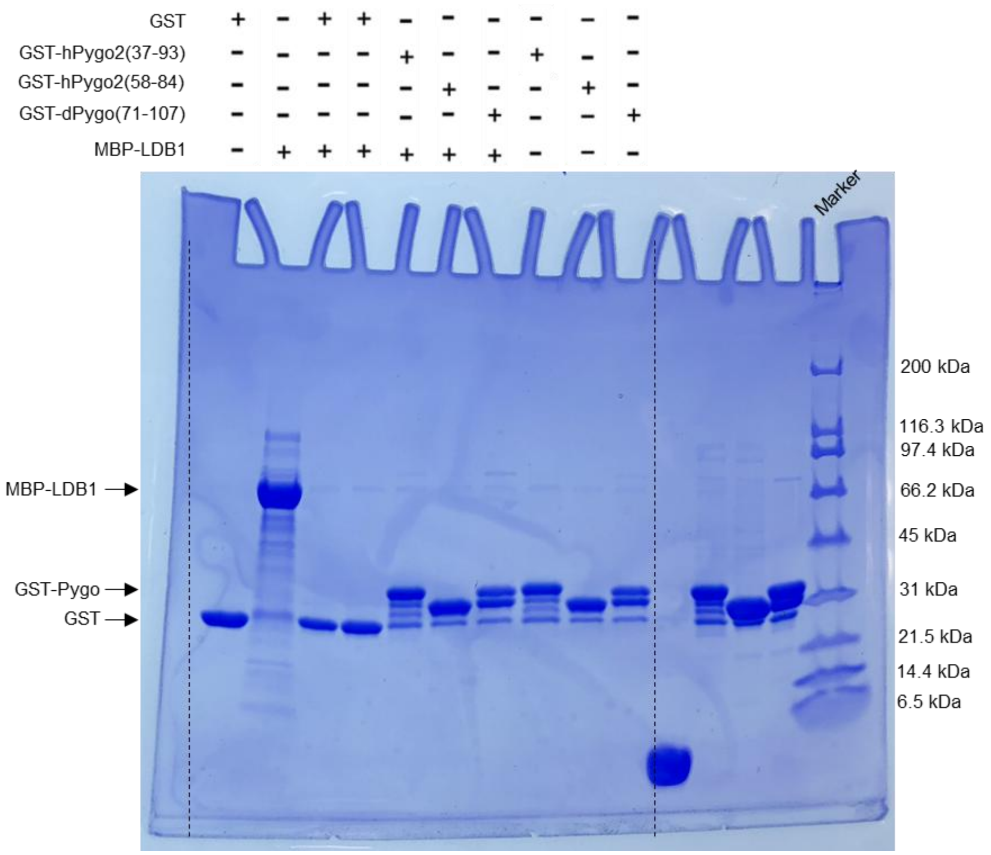

Supplementary Figure 3a

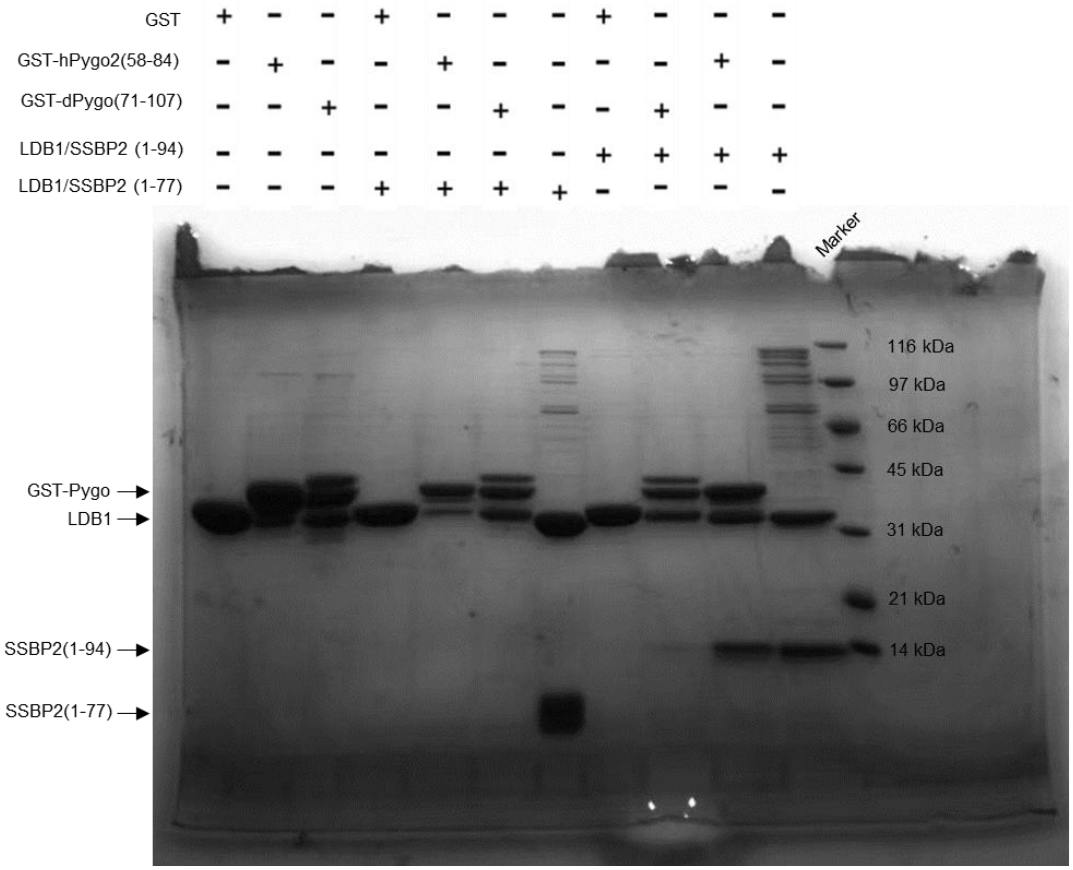

Supplementary Figure 3b

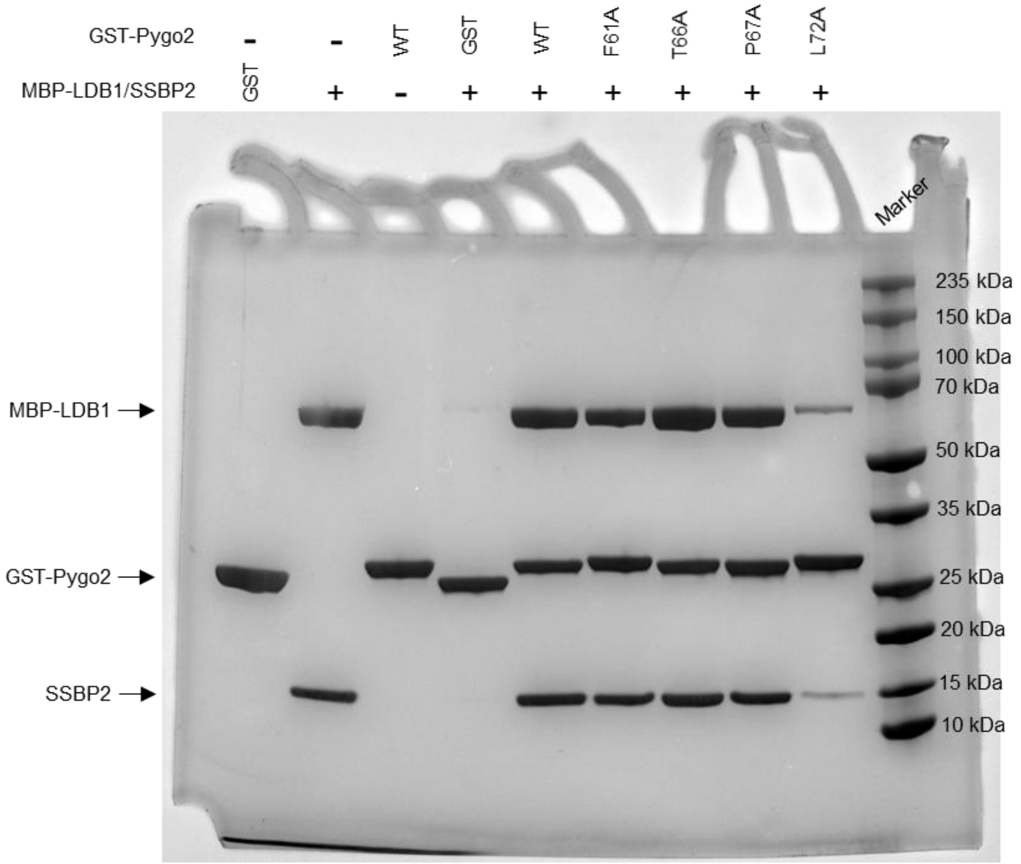

Supplementary Figure 6

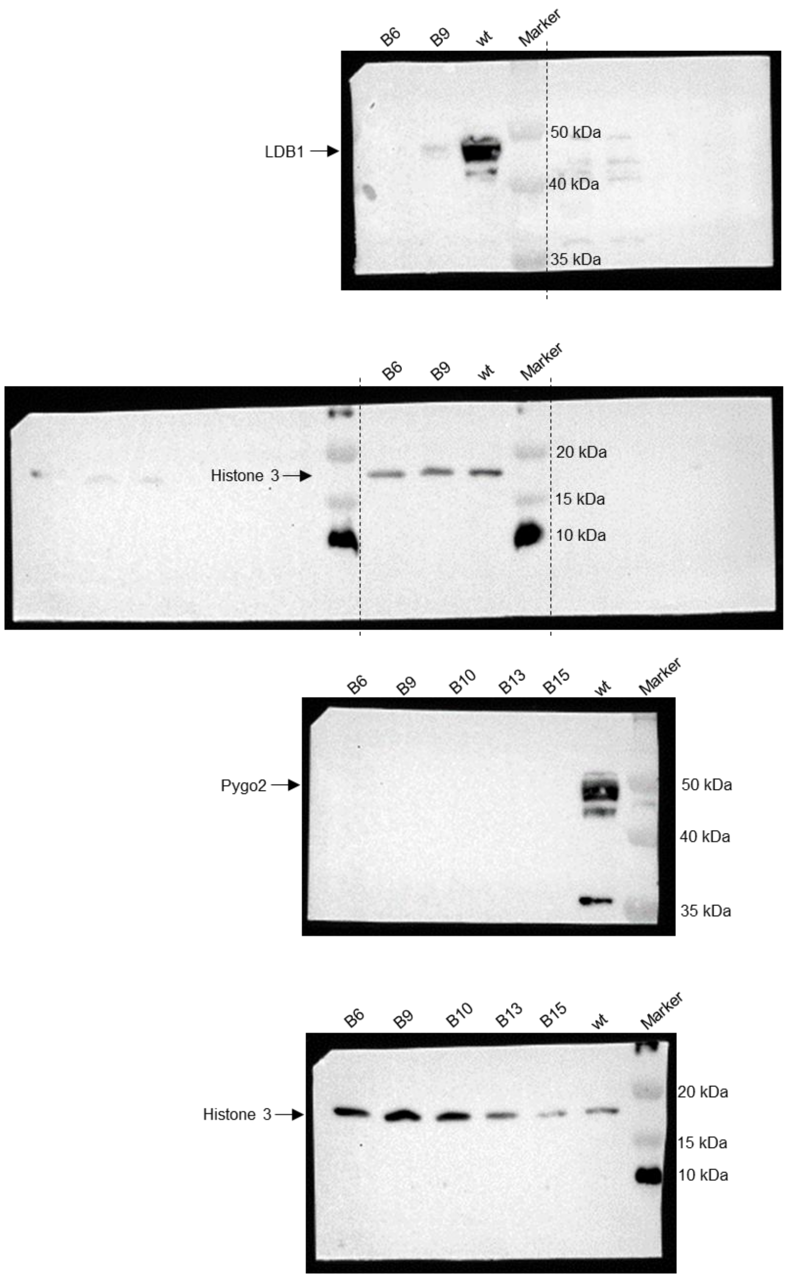

Supplementary Figure 8b
