# Supplementary material for: Structural basis of the interaction between BCL9-Pygo and LDB-SSBP complexes in assembling the Wnt enhanceosome
Source: Nat Commun. 2023 Jun 22;14:3702. doi: 10.1038/s41467-023-39439-9 (PMC10287724; doi:10.1038/s41467-023-39439-9)
Supplement: Supplementary file 3 — Description of Additional Supplementary Files [file 41467_2023_39439_MOESM3_ESM.pdf]

Description of Additional Supplementary Files

File Name: Supplementary Data 1

Description: All genes that are affected by Pygo DKO or LDB DKO in a statistically significant fashion (i.e. genes exhibiting >1.5 fold change in their expression).
